# The complete chloroplast genome of *Houttuynia cordata* Thunb. (Family: Saururaceae)

**DOI:** 10.1080/23802359.2019.1688105

**Published:** 2019-11-12

**Authors:** Feng Yu, Ya Liu, Renyi Zhang

**Affiliations:** School of Life Sciences, Guizhou Normal University, Guiyang, China

**Keywords:** *Houttuynia cordata*, chloroplast genome, phylogenetic relationship

## Abstract

The chloroplast genome sequence of *Houttuynia cordata* has been characterized from Illumina pair-end sequencing. The complete chloroplast genome was 161,090 bp in length, containing a large single-copy (LSC) region of 88,180 bp and a small single-copy (SSC) region of 19,204 bp, which were separated by a pair of 26,853 bp inverted repeat regions. Furthermore, phylogenetic analysis revealed that *H. cordata* is a sister of *Piper cenocladum*.

*Houttuynia cordata* Thunb. is a perennial herb found throughout many Asian countries including China, India, Korea, and Japan (Editorial Committee of Flora of China, Chinese Academy of Sciences 1999). It has important medicinal and edible value. However, the complete chloroplast genome has not been reported. Here, we determined the complete chloroplast genome sequence of *H. cordata* to provide genetic and genomic information to promote its breeding and systematics research.

In this study, *H. cordata* was sampled from Zhenning County, Anshun City, China (105°36′42″E, 26°2′37″N). The voucher specimen (GZNUYF201905001) was deposited in the herbarium of School of Life Sciences, Guizhou Normal University. The total genomic DNA was extracted from fresh leaves using Rapid Plant Genomic DNA Isolation Kit and sequenced based on the Illumina pair-end technology. The filtered reads were assembled using the program NOVOPlasty (Dierckxsens et al. 2017). The assembled chloroplast genome was annotated using PGA-Plastid Genome Annotator (Qu et al. 2019). The accurate new annotated complete chloroplast genome was submitted to GenBank with accession number MN475921.

The complete chloroplast genome of *H. cordata* is 161,090 base pairs (bp) in length, containing a large single-copy (LSC with 88,180 bp) region, a small single-copy (SSC with 19,204 bp) region, and two inverted repeat (IR with 26,853 bp) regions. The new sequence possesses total 133 genes, including 88 protein-coding genes, 37 tRNA genes, and 8 rRNA genes. Among all of these genes, four rRNA genes (i.e. 4.5S, 5S, 16S, and 23S rRNA), seven protein-coding genes (i.e. ndhB, rpl2, rpl23, rps12, rps7, ycf15, and ycf2), and seven tRNA genes (i.e. trnA-UGC, trnI-CAU, trnI-GAU, trnLCAA, trnN-GUU, trnR-ACG, and trnV-GAC) occur in double copies. The overall GC content of *H. cordata* chloroplast genome is 38.4%, while the corresponding values of the LSC, SSC, and IR regions are 36.8%, 32.3%, and 43.0%, respectively.

To infer the phylogenetic position of *H. cordata*, a maximum-likelihood (ML) tree was constructed using 70 shared protein-coding genes of *H. cordata* and 12 angiosperms plant taxa (Figure 1). The ML inference was performed using IQ-TREE (Nguyen et al. 2015) in PhyloSuite (Zhang et al. 2018) with 10,000 bootstrap replicates. The ML tree showed that *H. cordata* and *Piper cenocladum* were found to be a monophyletic group (Figure 1).

**Figure 1.**
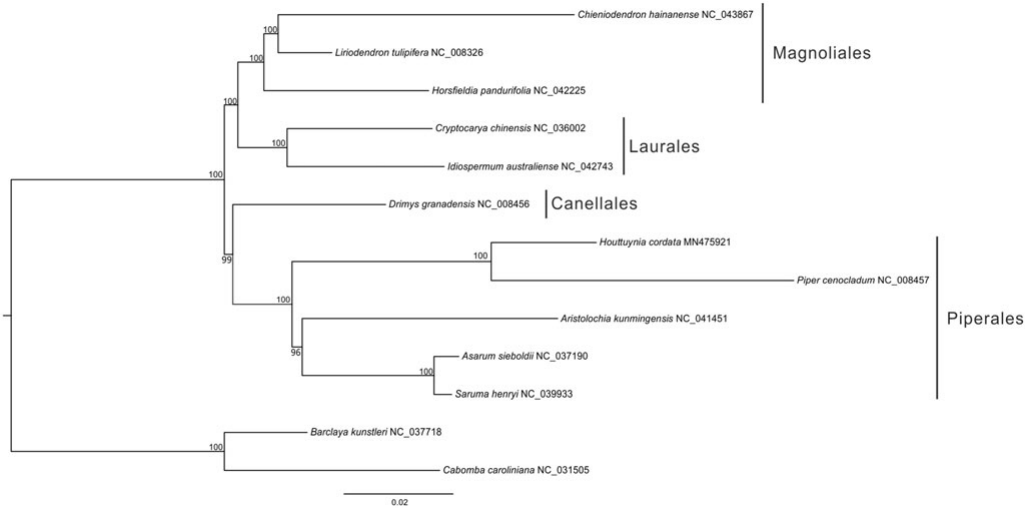
A maximum-likelihood (ML) tree inferred from 70 plastome genes is shown. Values along the branches represent ML bootstrap values.
